# Long-Lasting Input-Specific Experience-Dependent Changes of Hippocampus Synaptic Function Measured in the Anesthetized Rat

**DOI:** 10.1523/ENEURO.0506-18.2019

**Published:** 2019-09-05

**Authors:** Eliott R. J. Levy, Kally C. O’Reilly, André A. Fenton

**Affiliations:** 1 Center for Neural Science, New York University, New York, NY 10003; 2 Neuroscience Institute at the New York University Langone Medical Center, New York, NY 10016; 3 Department of Physiology, the Robert F. Furchgott Center for Neural and Behavioral Science, SUNY Downstate Medical Center, Brooklyn, NY 11203

**Keywords:** active place avoidance, conditioning, experience, hippocampus, memory, synaptic circuit function

## Abstract

How experience causes long-lasting changes in the brain is a central question in neuroscience. The common view is that synaptic function is altered by experience to change brain circuit functions that underlie conditioned behavior. We examined hippocampus synaptic circuit function *in vivo,* in three groups of animals, to assess the impact of experience on hippocampus function in rats. The “conditioned” group acquired a shock-conditioned place response during a cognitively-challenging, hippocampus synaptic plasticity-dependent task. The no-shock group had similar exposure to the environmental conditions but no conditioning. The home-cage group was experimentally naive. After the one-week retention test, under anesthesia, we stimulated the perforant path inputs to CA1, which terminate in stratum lacunosum moleculare (slm), and to the dentate gyrus (DG), which terminate in the molecular layer. We find synaptic compartment specific changes that differ amongst the groups. The evoked field EPSP (fEPSP) and pre-spike field response are enhanced only at the DG input layer and only in conditioned animals. The DG responses, measured by the population spiking activity and post-spike responses, are enhanced in both the conditioned and no-shock groups compared to home-cage animals. These changes are pathway specific because no differences are observed in slm of CA1. These findings demonstrate long-term, experience-dependent, pathway-specific alterations to synaptic circuit function of the hippocampus.

## Significance Statement

We investigated whether a hippocampus-dependent place avoidance memory causes long-lasting changes of hippocampus circuit function, as is commonly assumed. Immediately after testing one-week memory retention in rats, under anesthesia, the entorhinal cortex (EC) projection to dentate gyrus (DG) is strengthened, as estimated by the synaptic response to stimulation as well as the amplitude of the population action potential response. These changes are pathway specific because no differences are observed in the stratum lacunosum moleculare (slm) of CA1 where the EC projection also terminates. These findings of experience-dependent, pathway-specific alterations to synaptic circuit function in hippocampus are consistent with theories that posit that memory formation causes persistent alterations of neural circuit function.

## Introduction

How experience alters the brain to enable conditioned responses is an open question. The current view is that changes to synaptic circuit function underlie conditioned behavior. Changes to synaptic function have been difficult to identify and have focused on long-term potentiation (LTP) immediately after conditioning a behavior (Whitlock et al., 2006), although changes in synaptic function have also been observed during (Fernández-Lamo et al., 2018) or for one or more days after conditioning (Gruart et al., 2006; Park et al., 2015; Pavlowsky et al., 2017).

The two-frame active place avoidance task conditions a place response that depends on synaptic modifications in the dorsal hippocampus, specifically PKMζ-dependent and CaMKII-dependent LTP (Pastalkova et al., 2006; Tsokas et al., 2016; Hsieh et al., 2017; Rossetti et al., 2017). These studies have not, however, identified if the modifications to synaptic circuit function are universal or pathway-specific. In mice, following two-frame active place avoidance conditioning, robust changes were observed specifically in the CA3-CA1 synaptic response, but not in the entorhinal cortex (EC)-CA1 response, in *ex vivo* hippocampus slice recordings (Pavlowsky et al., 2017). In anesthetized mice, place avoidance training enhanced the excitability of granule cells in the dentate gyrus (DG) in response to EC stimulation (Park et al., 2015). Here, we extend these findings to the rat. Immediately after retraining one week after task acquisition, under anesthesia, we find training-induced changes in hippocampus function that are specific to the DG but not the CA1 targets of the EC inputs.

## Materials and Methods

All methods complied with Public Health and Service Policy on Humane Care and Use of Laboratory Animals and were approved by New York University Animal Welfare Committee, which follow National Institutes of Health guidelines.

### Animals

Twenty-two male Long–Evans (Charles River) rats arrived at the New York University animal facilities and were given at least a week to acclimate. The rats had free access to food and water and were single housed.

### Two-frame active place avoidance

Sixteen rats were used for the behavioral experiments. Rats were handled ∼5 min/d for 5 d before behavioral training. The training took place on an 81-cm diameter circular disk-shaped rotating arena with transparent walls (Bio-Signal Group). The arena could be stationary or rotate at 1 rpm. The position of a rat on the arena was monitored at 30 frames per second from an overhead camera using video-tracking software (Tracker, Bio-Signal Group). The software could track the rat’s position in the room as well as on the rotating arena and could deliver a mild constant current foot shock (0.3 mA, 60 Hz 500 ms) whenever the rat was detected in a 60° sector that was designated the shock zone. Although the arena rotated, the shock zone was stationary and was defined by stationary landmarks that were fixed in the room.

**Figure 1.**
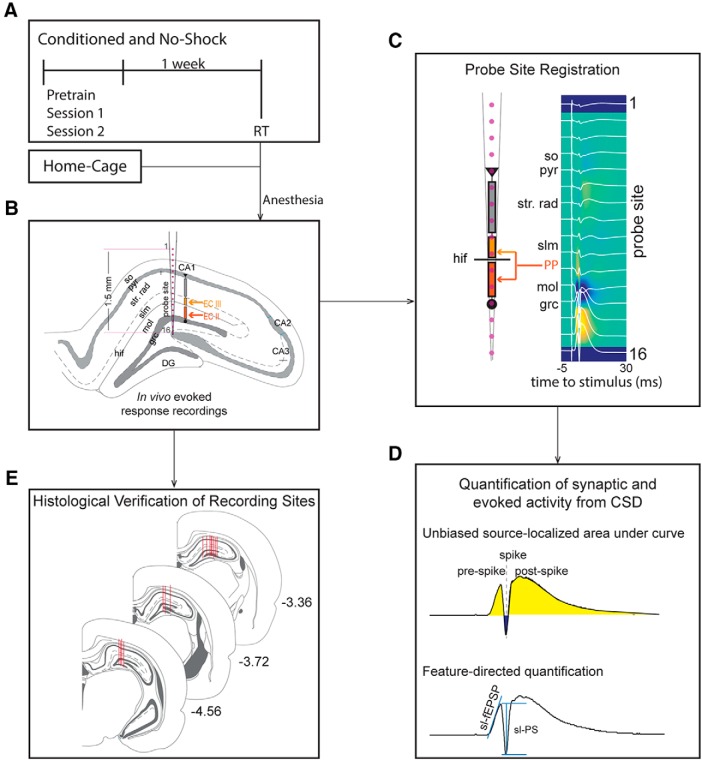
Evoked response paradigm. ***A***, Immediately after the retention trial, the conditioned and no-shock rats were anesthetized and underwent surgery for the evoked potential experiments. Home-cage rats were never handled before the evoked response recordings. ***B***, A 16-site linear electrode array was placed in the dorsal hippocampus, spanning the somatodendritic axis of CA1 and the DG. A bundle of stimulating electrodes was placed in the perforant path to stimulate EC inputs. ***C***, The recording probes were localized by performing CSD analysis, which identifies sinks and sources of the stereotypical response to perforant path stimulation at each layer. CA1: so = stratum oriens, pyr = pyramidal cell layer, strat. rad. = stratum radiatum, DG: mol = molecular layer, grc = granule cell layer, hif = hillar fissure. ***D***, Quantification of the synaptic and evoked activity from the CSD was performed either by calculating the AUCs for sinks and sources before or after the PS activity and was performed for each layer of CA1 and DG. The other approach quantified the fEPSP of the molecular and granule cell layers and population spiking activity of the granule cell layer. ***E***, Recording sites were verified histologically. Red lines indicate positions of the recording probes for the experiments performed. Anteroposterior coordinates relative to bregma are indicated next to the coronal sections.

The rats were given two 10-min pre-training trials to habituate to the stationary arena. During the two training days (sessions 1 and 2), the rats were given eight 10-min trials per day on the rotating arena. Rats in the conditioned group (*n* = 8) received shocks in the shock zone and rats in the no-shock group (*n* = 8) experienced the same physical conditions, except they were never shocked. The time between trials was ∼10 min. One week later, the rats were all returned to the rotating arena for 10 min to test memory retention. The shock was on in the shock zone for rats in the conditioned group. Immediately after the one-week experience the rats were anesthetized and prepared for assessing *in vivo* hippocampus evoked responses. The home-cage (*n* = 6) group consisted of rats that were never exposed to the behavioral room and were not handled (Fig. 1*A*).

### Behavioral assessment

Behavioral end-point measures were computed from the position timeseries using TrackAnalysis (Bio-Signal Group). We evaluated the distance walked on the arena, the number of entrances made into the shock zone, the time spent in each quadrant, and the time to enter the shock zone for the first time. The distance walked on the arena assesses locomotion. The shock zone entrances and the time in each quadrant estimate place avoidance and preference. The time to first enter the shock zone estimates avoidance memory across sessions.

### *In vivo* hippocampus-evoked responses

#### Surgical protocol

Immediately after the one-week retention trial, rats were anesthetized with 1.2 g/kg urethane (intraperitoneal) and placed in a stereotaxic frame. A 16-site linear-array silicon electrode with 30-μm diameter recording contacts and 100-μm spacing (Neuronexus; p/n: A 1 × 16 - 5mm-100-703) was placed in the dorsal hippocampus to span the somatodendritic axis of dorsal CA1 and DG (Fig. 1*B*). A six-wire stimulating electrode bundle was placed in the ipsilateral perforant path. The stimulating electrodes were made by twisting six nichrome wires (75 μm) together. The wires were cut at an angle to allow the six sites to span 1 mm. The following coordinates were used to target the perforant path fibers: relative to bregma, anteroposterior (–7.6 mm), mediolateral (4.1 mm), and dorsoventral (2.5–3.0 mm). The two-wire bipolar combination that evoked the largest response was selected for the stimulation experiments. A constant current stimulus isolation unit (WPI; model: A365RC) was used to deliver individual 150-μs stimulus pulses across the electrode pair.

The response to stimulation was recorded using Axona USBdacq software and the Axona hardware that was optimized for recording evoked potential responses. An attenuating resistor (either 18 or 47 kΩ) was connected to the Axona input to maximize the effective number of bits (16) used for digitization. The signals were low-pass filtered <5 kHz and digitized at 48 kHz.

#### Localization of the recording electrodes

Current source density (CSD) analysis was performed for source localization, minimizing volume conducted signals in the analyzed data. The CSD is computed from the second spatial derivative of the voltage along the recording depth:

$$CSD\left(z\right)=-\frac{V\left(z-\text{Δ}z\right)+V\left(z+\text{Δ}z\right)-2*V\left(z\right)}{\text{Δ}z^{2}}$$

where *CSD* (z) is the CSD at depth z, V (z) is the Voltage at depth z and △z = 100 μm is the distance between adjacent recording sites. The CSD is calculated in units of mV/μm^2^ and can be multiplied by a conductivity constant σ to measure it in units of A/μm^3^. By convention, CSD is computed as the negative of the second spatial derivative with negative CSD indicating an extracellular current sink and positive CSD an extracellular current source. Because it is isotropic (Hrabetová, 2005), we assume that the conductivity is homogeneous within the hippocampus as it was shown that variations in the conductivity had little effect on hippocampus CSD estimates (Holsheimer, 1987). Similarly, the conductivity was assumed to be similar across the groups.

The pattern of sinks and sources in each animal was used to localize the electrode recording sites to each somatodendritic location for an individual rat (Fig. 1*C*) as previously described (Brankack et al., 1993; Wu and Leung, 2003). Briefly, the DG granule cell layer was identified by the largest source in the lower channels that also included a sink, which was the population spike (PS) activity. The molecular layer was identified as the largest sink one to two channels above the granule cell layer. The stratum lacunosum moleculare (slm) was identified by two channels: the large sources above the molecular layer and an early latency sink in the one to two channels just above the largest source. Stratum radiatum was identified as a late latency sink and the pyramidal layer was identified as a late source above stratum radiatum. Stratum oriens was identified as the sink above the pyramidal layer. Based on the sinks and sources from one animal from the conditioned group and two animals from the no-shock group, the recording and/or stimulation electrodes did not appear to be properly placed and these three animals were excluded from the physiology analyses. Thus, for the physiology data, the final group numbers were conditioned *n* = 7, no-shock *n* = 6, home-cage *n* = 6.

#### Analysis of the responses evoked by stimulation

All analyses were performed offline using custom MATLAB software. We performed all estimates of the evoked responses on the CSD traces to minimize the impact of volume conduction. Accordingly, we refer to these evoked responses as source-localized (sl). Input-output (I-O) curves were generated by measuring the evoked responses to stimulus intensities ranging from 100 to 1000 μA in 100-μA steps. At each stimulus intensity, four voltage responses were recorded and the CSD computed for each response. All measurements were performed on each of the four responses and then averaged. Spike times were estimated at the DG-granule cell layer for each rat.

To visualize the responses along the somatodendritic axis, we aligned all recordings to the DG granule cell layer compartment and computed the group average CSD response to 800-μA stimulation for each rat. We chose to compute the average at 800 μA because that was the highest stimulation intensity delivered to all animals. The CSDs obtained at 800 μA were then averaged for each group to visualize the group-specific sinks and sources. The data were plotted with interpolation across adjacent electrodes using the *shading interp* MATLAB function.

##### Unbiased quantification: area under the curve (AUC)

We computed the AUC of the source localized (sl-) evoked response as an unbiased estimate of the response since it includes both direct and polysynaptic responses as well as spiking. The source-localized AUC (sl-AUC) was measured before and after population spiking at each compartment and separately for sources and sinks (Fig. 1*D*). Analysis of the individual sources and sinks within the CSD profile allowed us to quantify specific components of the response, such as the post-spike activity, that is otherwise difficult to identify and measure as it is comprised of excitatory and inhibitory responses. We note that interpreting an (inward) current sink or (outward) current source depends on knowing the functional anatomy of the relevant sites because active sinks and sources are associated with passive return sources and sink currents, respectively.

##### Feature-directed quantification

The evoked response contributions to the field EPSP (fEPSP) and to the PS are well-characterized and commonly used to estimate the responses of DG to entorhinal stimulation. Feature directed analysis was performed on the evoked CSD traces to measure the source-localized PS (sl-PS) and fEPSP (sl-fEPSP; Fig. 1*D*). The sl-PS was measured in the granule cell layer of the DG and two measures of the sl-fEPSP were made: the positive slope in the granule cell layer and the negative slope in the molecular layer. The time window for estimating sl-fEPSP was 1.5 ms after the stimulus was delivered until 1 ms before population spiking activity. This time restriction was chosen to prevent the inclusion of (variable) dendritic spiking activity (Herreras, 1990).

#### Boltzmann function fits

We examined fEPSP and PS (E-S) coupling in the DG by fitting a Boltzmann function to the data using the following equation for the *sl-PS* as a function of the *sl-fEPSP*:

$$s\mathrm{l-P}S=\frac{s\mathrm{l-P}S_{max}}{1+\exp\left(\frac{s\mathrm{l-f}EPSP_{50}-s\mathrm{l-f}EPSP}{S}\right)}$$

Where *sl-PS_max_* is the maximum *sl-PS*, *sl-fEPSP_50_* is the *sl-fEPSP* associated with the 50% *sl-PS_max_* response, and *S* is the slope (the slope being steeper as *S* decreases). The equation was fit for each animal and the *sl-PS_max_, sl-fEPSP_50_* and slopes were compared among the groups.

#### Paired-pulse inhibition

Stimulation to assess paired-pulse inhibition was performed at 65% of the intensity required to elicit the maximal sl-PS response. Stimulus pairs were delivered at increasing interstimulus intervals between the first and second pulses (5, 10, 20, 40, 80, 160, 320, and 640 ms). We allowed 30 s between each pair of stimuli. At each interstimulus interval, four responses were recorded and CSD analysis was performed to attenuate effects of volume conduction. The responses were measured and the ratio of the second to first response was used to estimate the amount of inhibition on the second stimulation due to the initial stimulation. These ratios were averaged across the four recordings.

#### Verification of stimulation and recording sites

At the end of the recordings, the rats were transcardially perfused with 1× PBS followed by 10% formalin. The brains were extracted and stored in formalin overnight and stored in 30% sucrose in 1× PBS until they were cut on a cryostat (40 μm) and thaw mounted onto gelatin-coated slides. The sections were dried overnight at room temperature and then Nissl stained. The slides were scanned at 10× with an Olympus VS120 microscope and the images were subsequently examined for electrode tracks to verify the stimulation and recording locations (Fig. 1*E*).

#### Statistical analysis

Statistical analysis was performed in JMP 14 (SAS). Behavioral data from all the animals were included in the analysis (conditioned: *n* = 8; no-shock *n* = 8). Multivariate ANOVA (MANOVA) was conducted for each behavioral phase (pretraining, training, retention) separately. For the pretraining and training phases, we conducted MANOVA analyses with the trials used as a repeated measure; Hotelling’s trace correction was used when sphericity was violated. Additionally, when relevant, two-group comparisons were made using Student’s *t* tests, with degrees of freedom adjusted for unequal variances. Electrophysiological data were assessed by ANOVA to identify statistically significant group, stimulation intensity, and group × stimulation intensity differences between conditioned, no-shock, and home-cage rats. Stimulation intensity was treated as a continuous variable. Tukey’s honest significant difference (HSD) *post hoc* tests evaluated pair-wise differences. The effect size η^2^ is given when a non-significant statistical trend is observed. Statistical significance was set at *p* < 0.05.

## Results

### Conditioned behavior

**Figure 2.**
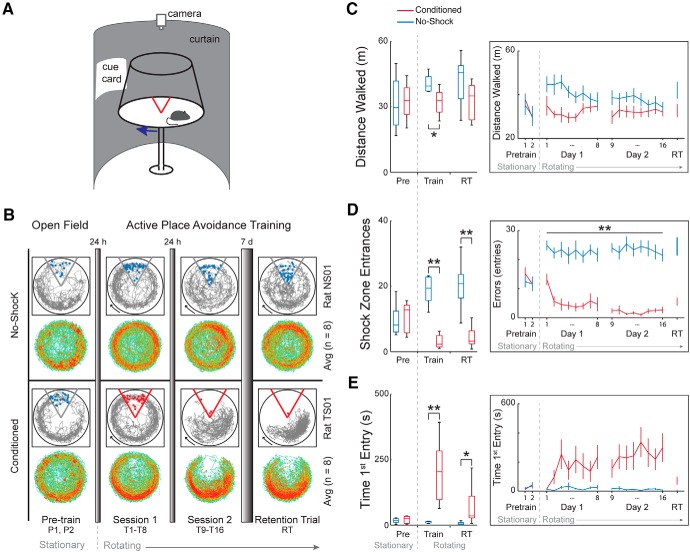
Memory-related behavior is different between conditioned and no-shock rats. ***A***, Rats were placed on a rotating arena and conditioned to avoid a mild foot shock within a 60**°** sector that was stationary within the room. The no-shock group was exposed to the same conditions, but without foot shock. ***B***, The rats were given two pretraining trials on a stationary arena without shock. The next day the rats started 2 d of conditioning (sessions 1 and 2). Each day a conditioning session consisted of eight training trials. One week after the end of conditioning, the rats were given a retention trial to test memory. For the conditioned group, the retention trial was conducted with the shock on. All trials were 10 min with at least 10 min intertrial intervals. Example paths (in gray) of one no-shock rat and one conditioned rat are shown over the course of training. The red dots represent where the animal received shocks and blue dots indicate where the animal would have received shock if the shock had been present. The heat maps show average time spent for all rats in each 0.3 × 0.3-cm region for each group. Red represents an average dwell of >0.017 s. By the end of the training period, the conditioned rats avoid the 60**°** sector (bottom) while the no-shock rats do not exhibit a place bias. ***C–E***, Box plots of behavioral measures for the pre-training session, the training sessions (trials 1–16), and the retention trial show there are differences between no-shock and conditioned rats. Asterisks in the box plots (***C–E***, left) indicate a main effect of group, while asterisks in the line graph (***C–E***, right) indicate group differences. no-shock, *n* = 8; conditioned, *n* = 8. Plots to the right of the boxplots represent mean ± SEM on a trial-by-trial basis; *p < 0.05, **p < 0.01.

The arena is stationary during the pretraining trials, allowing the assessment of locomotor behavior, similar to that done in an open field test. Because the rats were randomly assigned to the conditioned and no-shock groups, we did not expect to see group differences in locomotor activity on the stationary arena. Indeed, before training, the conditioned and no-shock control groups are identical in their exploratory activity (Fig. 2*B*). The average time spent in each location for all rats in each group (Fig. 2*B*, heat maps), with example traces of a rat from each group (Fig. 2*B*, gray traces), indicates that during pretraining, rats do not avoid going into the region that will eventually be designated as the shock zone. However, to investigate biases for locations on the arena, we analyzed the proportion of time spent in each quadrant. There is a quadrant effect (*F*_(3,56)_ = 3.12, *p* = 0.03) because rats show a preference to spend time in at least one quadrant, but the group interactions are not significant, indicating no influence of group (group × quadrant: *F*_(3,56)_ = 0.41, *p* = 0.74; group × quadrant × trial: *F*_(3,56)_ = 0.16, *p* = 0.92), and neither is the trial × quadrant interaction significant, indicating steady state preferences (*F*_(3,56)_ = 0.82, *p* = 0.49). Although it appears from the heat maps (Fig. 2*B*) that the animals are spending more time in the quadrant opposite the eventual shock zone, *post hoc* analysis on the quadrants indicates no preference for this specific quadrant. Furthermore, the two groups are indistinguishable in their behaviors during this initial exploratory period (Fig. 2*C–E*) in the total distance walked on the stationary arena (group: *F*_(1,14)_ = 0.04, *p* = 0.85; trial: *F*_(1,14)_ = 10.52, *p* = 0.006; group × trial: *F*_(1,14)_ = 0.78, *p* = 0.39), the number of times they enter the eventual shock zone (group: *F*_(1,14)_ = 0.23, *p* = 0.64; trial: *F*_(1,14)_ = 2,27, *p* = 0.15; group × trial: *F*_(1,14)_ = 0.95, *p* = 0.34) and the latency to first enter the eventual shock zone (group: *F*_(1,14)_ = 8 × 10^−4^, *p* = 0.98; trial: *F*_(1,14)_ = 4.77, *p* = 0.05; group × trial: *F*_(1,14)_ = 0.55, *p* = 0.47).

Conditioning the rats to avoid the location of the shock during the training trials results in multiple behavioral differences. Conditioned rats learn to avoid the location of shock on the rotating arena in the two-frame active place avoidance task whereas rats exposed to the same environment without shock (no-shock rats) do not express a conditioned place response (Fig. 2*B*). During training, an effect of quadrant is observed (*F*_(3,112)_ = 10.53, *p* = 10^−6^) as well as a group × quadrant interaction (*F*_(3,112)_ = 26.34, *p* = 10^−13^) and a group × trial × quadrant effect (*F*_(21,215.08)_ = 1.7, *p* = 0.032), but no other interaction (trial × quadrant effect: *F*_(21,215.08)_ = 1.54, *p* = 0.067; day × quadrant effect: *F*_(3,112)_ = 0.20, *p* = 0.90; group × day × quadrant effect: *F*_(3,112)_ = 0.65, *p* = 0.58; trial × day × quadrant effect: *F*_(21,215.08)_ = 1.37, *p* = 0.13; group × trial × day × quadrant effect: *F*_(21,215.08)_ = 1.32, *p* = 0.17). *Post hoc* tests done separately for each group during training show an effect of sector for both groups but with a preference for the opposite sectors: Trained animals spend the least time in the shock quadrant while no-shock animals spend the most time there. Both groups of rats appear to have an increase in distance walked early in session 1 compared to what is observed in pretraining, which may be due to the novelty of being on a rotating arena that was previously stationary. As seen in the example trajectory and the group-averaged time spent in each location on the arena in the last training trial of session 2 (Fig. 2*B*), the conditioned rats learn to avoid the shock zone. The conditioned rats walk 18% less than the no-shock rats during training (Fig. 2*C*), which is because of the no-shock rats’ increased locomotion, and due to the restriction of the conditioned rats walking in only three of the four quadrants (distance walked; group: *F*_(1,28)_ = 9.76, *p* = 4.12 × 10^−3^; day: *F*_(1,28)_ = 0.82, *p* = 0.37; trial: *F*_(7,22)_ = 1.57, *p* = 0.20; group × day: *F*_(1,28)_ = 0.67, *p* = 0.42; group × trial: *F*_(7,22)_ = 1.4, *p* = 0.26; day × trial: *F*_(7,22)_ = 1.26, *p* = 0.31; group × day × trial: *F*_(7,22)_ = 0.60, *p* = 0.75). The conditioned group quickly reduces the number of entries into the shock zone while the no-shock rats continue to enter the shock zone location throughout the entire training period (shock zone entrances; group: *F*_(1,28)_ = 171.12, *p* = 10^−13^; day: *F*_(1,28)_ = 0.97, *p* = 0.33; trial: *F*_(7,22)_ = 1.50, *p* = 0.22; group × day: *F*_(1,28)_ = 2.04, *p* = 0.16; group × trial: *F*_(7,22)_ = 2.55, *p* = 0.044; day × trial: *F*_(7,22)_ = 0.7, *p* = 0.67; group × day × trial: *F*_(7,22)_ = 0.65, *p* = 0.71; *post hoc* Student’s *t* tests to evaluate group differences at each trial are all significant at *p* < 0.05; Fig. 2*D*). Conditioned rats also show avoidance memory by increasing their latency to enter the shock zone over the course of conditioning (time to first entry; group: *F*_(1,28)_ = 29.57, *p* = 10^−6^; day: *F*_(1,28)_ = 1.43, *p* = 0.24; trial: *F*_(7,22)_ = 0.84, *p* = 0.56; group × day: *F*_(1,28)_ = 1.91, *p* = 0.18; group × trial: *F*_(7,22)_ = 1.60, *p* = 0.19; day × trial: *F*_(7,22)_ = 0.36, *p* = 0.92; group × day × trial: *F*_(7,22)_ = 0.14, *p* = 0.99; Fig. 2*E*).

The impact of the training experience on behavioral measures persists for at least one week following the end of conditioning. During the retention trial, an effect of quadrant (*F*_(3,56)_ = 4.49, *p* = 6.80 × 10^−3^) and a group × quadrant interaction is still observed (*F*_(3,56)_ = 9.36, *p* = 10^−5^). Again, *post hoc* tests done separately on each group during the retention trial show that conditioned rats spend the least amount of time in the shock quadrant (*F*_(3,28)_ = 9.42, *p* = 1.82 × 10^−4^ with Tukey’s *post hoc* indicating preference for the quadrant opposite to the shock quadrant), which is also seen in the reduced number of entrances into the shock zone compared to no-shock rats (*t*_(9.81)_ = 5.95, *p* = 1.52 × 10^−4^). During the retention trial, the no-shock rats show no preference for any of the quadrants (*F*_(3,28)_ = 0.97, *p* = 0.42) and there is no significant group difference in the distance walked (*t*_(13,18)_ = 1.91, *p* = 0.078). In addition to displaying a preference for avoiding the shock quadrant, the conditioned rats have an increased latency to enter the shock quadrant (*t*_(7.09)_ = 2.42, *p* = 0.046), another indicator that the conditioned rats have a one-week-old place avoidance memory.

### Evoked potential results

**Figure 3.**
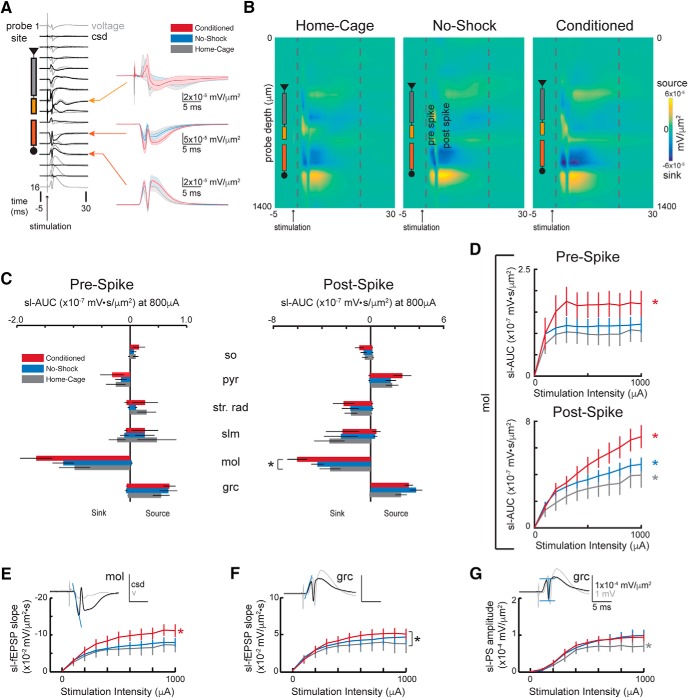
Behavior-induced alterations to DG circuit function assessed by response to perforant path stimulation. ***A***, The CSD (black traces) was computed from the recorded voltage traces (gray) to remove volume conducted signals. The sites determined to be located in slm, molecular layer and granule cell compartments (orange arrows) were used for further evaluation. The average ± SEM (shading) sl-CSD are shown. ***B***, Average CSD heatmaps were generated for each group. Cooler colors are sinks (inward current) and warmer colors are sources (outward current). The sink in the molecular layer appears to increase with experience and cognitive demand. ***C***, The AUC of the pre-spike response (1.5 ms after the stimulation was delivered until the spike occurred) and the post-spike response (from the spiking activity until 20 ms after stimulation) were quantified at the maximum stimulation given to all animals (800 μA) for each layer of the somatodendritic axis of the dorsal hippocampus. The pre-spike sink at the molecular layer is not changed by experience whereas the post-spike sink in the molecular layer is significantly altered by experience. CA1: so = stratum oriens, pyr = pyramidal cell layer, strat. rad. = stratum radiatum, DG: mol = molecular layer, grc = granule cell layer. ***D***, top, The I-O curve that characterizes the pre-spike sink, synaptic population response to the full range of stimulus intensities, is significantly increased in conditioned rats compared to both no-shock and home-cage groups. Bottom, The post-spike response to increasing stimulation intensity is significantly increased in conditioned rats compared to the no-shock response, which is also significantly increased compared to home-cage rats. ***E***, The sl-fEPSP characterizing the population synaptic response at the molecular layer is significantly increased by conditioning, confirming the findings based on the sl-AUC. ***F***, The sl-fEPSP at the granule cell layer is significantly different between conditioned and home-cage groups. ***G***, The PS response at the granule cell layer is significantly increased by experience. Example CSD waveforms are plotted in ***E–G*** in black for illustration with schematization of the measurement corresponding to the figure. The voltage trace is underlaid in gray. Home-cage, *n* = 6; no-shock, *n* = 6; conditioned, *n* = 7. Data are mean ± SEM. *p < 0.05. Colored asterisks indicate which group is different from the other two.

We examined synaptic function in the dorsal hippocampus under anesthesia immediately following the retention trial. As is seen by the overlapping traces of the voltage and the CSD response waveforms (Fig. 3*A*), performing CSD analysis reduces the influence of volume conduction on the waveforms and is therefore a better localized estimate of the evoked activity at the different somatodendritic compartments of CA1 and DG. We registered the data to the somatodendritic locations and then calculated group average CSD traces (Fig. 3*B*), which appear different, although we did not further analyze the spatial profile of the CSD.

All estimates of evoked activity were performed on the CSD-corrected responses and are referred to as source-localized. To evaluate the stimulation responses, we chose to take two separate approaches, a feature-directed quantification and an unbiased evaluation of the evoked responses. The first approach is to quantify specific features of the evoked response that are traditionally used to estimate the synaptic and spiking components, the slope of the evoked sl-fEPSP response and the amplitude of the sl-PS, respectively. The second approach evaluates the evoked activity localized to a single electrode at a specific location along the somatodendritic axis. Without relying on assumptions about the waveform of the response, we measured the AUC of the CSD trace at this site. The activity following spiking is particularly difficult to quantify using traditional methods but can be captured in a straightforward manner by measuring the AUC of the response that occurs after the PS. Whether this activity corresponds to active transmembrane currents or passive return currents cannot be determined from the CSD alone and requires knowledge of the functional anatomy for a complete interpretation, and we therefore analyzed the CSD source and sink components separately. In contrast, the activity preceding the PS standardly estimated by the slope of the fEPSP can also be estimated by measuring the AUC before the PS, which quantifies the entire response instead of the response at a specific time point; these two estimates of the synaptic response at the molecular layer are correlated in our dataset (*r* = 0.87, *p* = 10^−57^).

To get an initial sense of where training-induced changes in the evoked response might occur, we quantified the source-localized activity before and after the PS activity by measuring the sl-AUC in response to 800-μA stimulation, which is the maximum stimulation intensity that was recorded for all animals (Fig. 3*C*). Because we wanted to separately quantify the activity before and after the sl-PS, we first determined if the sl-PS times differed among the groups. While the sl-PS times do differ across the three groups (*F*_(2,89)_ = 3.72, *p* = 0.03) as well as by stimulation intensity (*F*_(1,89)_ = 9.68, *p* = 2.5 × 10^−3^), there is no interaction (*F*_(2,89)_ = 0.01, *p* = 0.99) and no *post hoc* group differences. While the pre-spike activity at the molecular layer is not different between groups (*F*_(2,16)_ = 2.05, *p* = 0.16, η^2^ = 0.20; Fig. 3*C*, left), the post-spike sink at the molecular layer is significantly different among the groups (*F*_(2,16)_ = 3.65, *p* = 0.0496). The difference between the conditioned and home-cage groups was confirmed by *post hoc* tests, but neither group is different from the no-shock group (Fig. 3*C*, right). Although the pre-spike molecular layer sink represents the perforant path input, it is uncertain to what extent this difference in the post-spike molecular layer sink reflects a training-induced difference in either an active transmembrane current or a passive return current.

We evaluated the I-O curves of pre- and post-spiking responses (sl-AUC) at the molecular layer (Fig. 3*D*), both of which show a significant effect of group due to greater statistical sensitivity of repeating the measurements for different stimulus intensities. The pre-spike response plateaus at ∼300-μA stimulation for all groups and is greater for the conditioned group (group: *F*_(2,180)_ = 17.38, *p* = 10^−7^; stimulation intensity: *F*_(1,180)_ = 2.93, *p* = 0.09; interaction: *F*_(2,180)_ = 0.39, *p* = 0.68; with *post hoc* differences between the conditioned group and the other groups; Fig. 3*D*, top). After the spike, the response increases with stimulation intensity and is different between the groups such that conditioned > no-shock > home-cage (group: *F*_(2,180)_ = 23.92, *p* = 10^−10^; stimulation intensity: *F*_(1,180)_ = 99.25, *p* = 10^−19^; interaction: *F*_(2,180)_ = 6.04, *p* = 0.003; with *post hoc* differences between all groups; Fig. 3*D*, bottom).

Traditionally, the early slope of the fEPSP is used to estimate the synaptic response and the PS amplitude is used to estimate synchronous neuronal discharge. We estimated these values from the CSD-corrected responses. The conditioned group sl-fEPSP at the molecular layer is larger than that of the no-shock and home-cage rats (group: *F*_(2,180)_ = 13.65, *p* = 10^−6^; stimulation intensity: *F*_(1,180)_ = 48.33, *p* = 6.41 × 10^−11^; interaction: *F*_(2,180)_ = 2.30, *p* = 0.10; conditioned > no-shock = home-cage; Fig. 3*E*).

**Figure 4.**
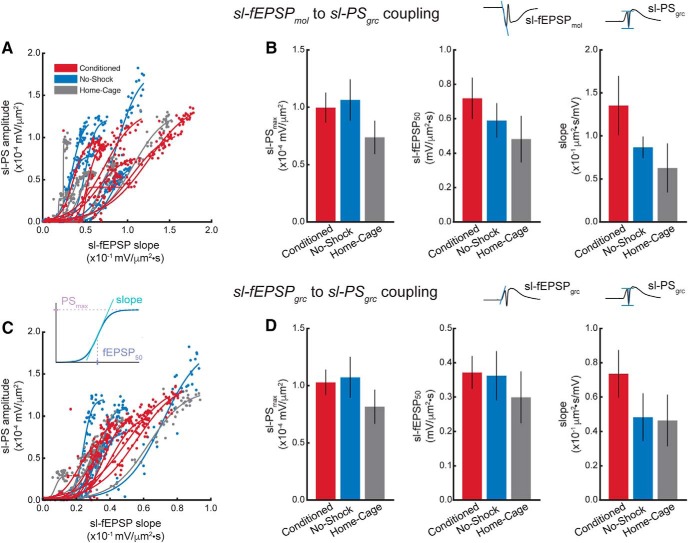
Coupling between the field synaptic potentials and population spiking is not different between groups. ***A***, The sl-fEPSP measured at the molecular layer is plotted against the sl-PS measured at the granule cell layer for each stimulation response, with intensity varying from 100 to 1000 μA. The Boltzmann fit to each animal’s E-S coupling is overlaid. ***B***, Average parameters of the Boltzmann fit per group show no difference due to experience in the sl-PS_max_ (left), the sl-fEPSP_50_ (middle), and the slope (right). ***C***, The sl-fEPSP measured at the granule cell layer is plotted against the sl-PS measured at the granule cell layer. ***D***, Average parameters of the Boltzmann fit per group also show no difference due to experience in the sl-PS_max_ (left), the sl-fEPSP_50_ (middle), and the slope (right). The inset in ***C*** schematizes the role of the parameters of the Boltzmann fit. The example CSD waveforms in ***B***, ***D*** are plotted for illustration.

Although the overall CSD responses (Fig. 3*C*) do not appear different among the groups at the granule cell layer, there is a group effect on both the sl-fEPSP slope (group: *F*_(2,180)_ = 3.79, *p* = 0.02; stimulation intensity: *F*_(1,180)_ = 35.23, *p* = 10^−8^; interaction: *F*_(2,180)_ = 0.79, *p* = 0.46; conditioned > no-shock = home-cage; Fig. 3*F*) and the sl-PS amplitude (group: *F*_(2,180)_ = 5.92, *p* = 0.003; stimulation intensity: *F*_(1,180)_ = 160.76, *p* = 10^−27^; interaction: *F*_(2,180)_ = 1.64, *p* = 0.20; conditioned = no-shock > home-cage; Fig. 3*G*). None of these parameters measured in the physiology correlate with the behavior, in particular with the time to first enter the shock zone on the retention trial (data not shown). These differences are also not accompanied by differences in E-S coupling [molecular layer sl-fEPSP to granule cell layer sl-PS (Fig. 4*A*,*B*): sl-PS_max_: *F*_(2,16)_ = 1.24, *p* = 0.32, η^2^ = 0.13; sl-fEPSP_50_: *F*_(2,16)_ = 1.00, *p* = 0.39, η^2^ = 0.11; slope: *F*_(2,16)_ = 1.82, *p* = 0.19, η^2^ = 0.19; granule cell layer sl-fEPSP to granule cell layer sl-PS (Fig. 4*C*,*D*): sl-PS_max_: *F*_(2,16)_ = 0.86, *p* = 0.44, η^2^ = 0.10; sl-fEPSP_50_: *F*_(2,16)_ = 0.36, *p* = 0.70, η^2^ = 0.04; slope: *F*_(2,16)_ = 1.18, *p* = 0.33, η^2^ = 0.13]. Although the slopes of the E-S coupling appear different (Fig. 4*B*,*D*, right panel), significance is not reached, likely due to the small effect sizes. Indeed, a power analysis with power set to 0.80 indicates three to four times larger sample sizes are needed to detect group differences in the E-S coupling. Finally, the differences in sl-fEPSP and sl-PS cannot not be easily attributed to changes in feedback inhibition, as there is no difference in paired-pulse inhibition measured as the ratio sl-PS2/sl-PS1 (group: *F*_(2,113)_ = 0.13, *p* = 0.88; ISI: *F*_(1,113)_ = 2.73, *p* = 0.10; interaction: *F*_(2,113)_ = 0.49, *p* = 0.61; data not shown). Nonetheless, other estimates of altered inhibition merit assessing (Ruediger et al., 2011).

## Discussion

### Summary

Here, we show that one week after two-frame active place avoidance conditioning, rats retain the conditioned place response and express an altered synaptic circuit response to perforant path stimulation of the EC input to DG. This indicates that learning the hippocampus-dependent place avoidance is associated with altered neocortical-hippocampal circuit function. The functional alteration of this neural circuit component is specific to the population synaptic component of the response localized to the molecular layer of DG where the stimulated pathway terminates, as observed in both the pre-spike sl-AUC (Fig. 3*D*) and the sl-fEPSP (Fig. 3*E*). No change is detected in lacunosum moleculare of CA1 where perforant path fibers also terminate. The change in synaptic circuit function of the entorhinal-to-DG pathway is not observed in no-shock control rats that experienced the identical physical conditions as the conditioned rats with one exception - they were never shocked. Because on average, the conditioned rats only enter the shock zone ∼5 times during each 10-min training session (Fig. 2*D*), the physical experience of the environment only differs between the conditioned and no-shock groups during shock, which comprised ∼2.5 s (5 × 500 ms) or <1% of each training session’s duration. While the conditioning is sufficient to alter population synaptic function, it also results in increased neuronal discharge, evidenced as an increased PS response at the granule cell layer, whereas the enhanced sl-fEPSP at the granule cell layer is harder to interpret (Fig. 3*F*) because the PP terminates at the molecular layer. Within the conditioned DG, the evoked responses are greater (Fig. 3*E–G*) and both the pre-spike population synaptic response and the post-spike population response are enhanced compared to the corresponding responses in home-cage DG (Fig. 3*D*). In contrast, the conditioning does not alter E-S coupling, indicating that synaptic input responses change, likely without an effective change in the corresponding excitability, and the changes are input specific. Some components of the evoked responses are altered in both the conditioned and no-shock groups while other components are altered only in the conditioned group. An enhanced synaptic component of the evoked response was only observed in the conditioned rats, whereas compared to home-cage rats, the measures of neuronal discharge and post-spike activity were enhanced after either the conditioned or non-conditioned experience. Perhaps these changes reflect learning because even the no-shock rats’ experience leads to spatial learning as measured by hippocampal physiology (Muller and Kubie, 1987; Wilson and McNaughton, 1993; Radwan et al., 2016).

The observed increases in sl-PS amplitude in the conditioned and no-shock groups compared to the home-cage group appears not to be due to increased intrinsic excitability as no significant effect of experience on E-S coupling is observed. However, E-S coupling reflects both intrinsic excitability and the balance of excitation/inhibition (Lu et al., 2000; Daoudal et al., 2002), which cannot be distinguished without additional experiments. Alternatively, either altered feedback or feedforward inhibition could account for the observations, but although the paired-pulse paradigm used in these studies did not detect a group difference, other estimates of feedback inhibition are warranted before concluding that feedback inhibition is unchanged by active place avoidance training. Nonetheless, because learning-induced changes in interneuron connectivity have been described in DG (Ruediger et al., 2011), and inhibition-sensitive increases of Schaffer collateral synapse effectiveness have been observed in conditioned mice (Pavlowsky et al., 2017), it may be the case that learning causes more complex neural circuit changes that might be obscured in the present study by use of anesthesia, which can differentially impact excitatory and inhibitory neurotransmission. The impact of the conditioning and no-shock experiences on feedforward inhibition requires further examination, ideally in awake, freely-behaving subjects.

### Relationship to prior work

Previous work in both awake rats (Whitlock et al., 2006) as well as *ex vivo* mouse hippocampus slices (Pavlowsky et al., 2017) observed changes in CA3-CA1 synaptic function after avoidance learning, whereas in the mouse slice experiments EC-CA1 synaptic responses were unchanged after place avoidance learning (Pavlowsky et al., 2017), similar to the conditioned rats in the present study. *In vivo* recordings of anesthetized mouse DG responses to stimulation of EC input showed an increased fEPSP response in trained versus naïve animals while the amplitude of the PS and the E-S coupling was unchanged (Park et al., 2015). While our results are similar to Park et al. (2015), in that we find that the sl-fEPSP is increased with conditioning, our results contrast with these previous results because we also see an impact of conditioning on the sl-PS amplitude. This discrepancy could be due to the differences in analyzing our signals; by recording at multiple, evenly spaced sites, we were able to reduce the influence of volume conduction and better localize our measurements by computing the CSD and making our measurements on the CSD waveforms. Consistent with prior work, we conclude that active place avoidance conditioning causes long-lasting changes to hippocampus circuit function in both mice and rats.

Other forms of conditioning have also been reported to change hippocampus circuit function. Forms of eyeblink conditioning cause LTP-like NMDAR- and PKMζ-dependent enhancement of the CA3-CA1 pathway, assessed in awake mice (Gruart et al., 2006; Gruart and Delgado-García, 2007; Madroñal et al., 2010). Non-aversive, non-associative learning, such as in an object recognition memory task was also associated with enhancement of CA3-CA1 input (Clarke et al., 2010). Changes in EC-DG synaptic responses were found over the course of operant conditioning, as were changes in EC-CA1. The changes were moderate in both cases and were behavior dependent. Remarkably, the EC-DG changes were only measurable when animals approached the lever, and these were only observed during the learning phase of the paradigm and returned to baseline after the task was acquired. Furthermore, changes in EC-CA1 were only significantly altered when animals were going to retrieve food, eating the food, or grooming (Fernández-Lamo et al., 2018). Although it appears that some of these changes are transient (Fernández-Lamo et al., 2018), other changes measured *in vivo* can persist for days (Park et al., 2015) and cannot be easily explained by a behaviorally-induced, temporary rise in brain temperature (Moser et al., 1993a,b; Moser, 1995). This is especially the case when we consider data from *ex* vivo slice experiments (Pavlowsky et al., 2017) and under constant conditions of anesthesia (Park et al., 2015), as in the present study. While the present findings unequivocally demonstrate that hippocampus-dependent learning is accompanied by persistent changes to hippocampus circuit function, changes in circuit function may not be unique to hippocampus and may be observable only in specific behavioral states (Fernández-Lamo et al., 2018). It is also not known whether the functional changes indicate LTP and other synaptic changes that, according to the synaptic plasticity and memory hypothesis, underlie long-term memory. Definitive evidence remains elusive (Takeuchi et al., 2014; Ryan et al., 2015; Tonegawa et al., 2015).

### Changes in synaptic function and synaptic plasticity

As described above, we, like others, have observed learning-related group differences in hippocampus circuit function that were detected *in vivo* by measurements of neural population activity such as the evoked responses of the present study. Because the groups only differ in their experience, we interpret those differences to indicate changes in circuit function consequent to different experiences that are our experimental manipulations. Such changes must be widespread if they are measurable in population activity and are thus at odds with expectations that memory-related functional changes are sparse (Whitlock et al., 2006; Takeuchi et al., 2014). Nonetheless they have been observed, but according to sparsity predictions, they are unlikely to simply reflect the LTP or depression of the specific synapses that store memory.

Here, we observed that some components of the evoked responses are altered in both the conditioned and no-shock groups while other components are altered only in the conditioned group, highlighting that behavioral history is important for interpreting estimates of neural circuit function, and that it can be difficult to definitively attribute measurable changes to specific changes in synaptic function (Moser et al., 1994; Andersen and Moser, 1995; Moser, 1995; Talbot et al., 2018). In particular, we observe altered sl-fEPSP and pre-spike activity in the conditioned group and altered sl-PS and post-spike activity in both the conditioned and no-shock groups. These changes in both the conditioned and no-shock rats could be explained by various mechanisms that have been observed after exposing rodents to enriched environments. In the current study, both the conditioned and no-shock rats were handled as well as exposed to a novel environment, which may serve as enrichment compared to the home-cage rats. The mechanisms by which enrichment induced neuronal alterations include increased neurogenesis (Kempermann et al., 1997, 2002), increased dendritic length (Faherty et al., 2003), increased spine density (Moser et al., 1994), increased synaptic density (Rampon et al., 2000), and increased synaptic protein expression (Frick and Fernandez, 2003; Nithianantharajah et al., 2004) to name a few. Given the impact of enrichment on these mechanisms, it is possible that pre-spike versus post-spike alterations are a generalizable feature of conditioning versus experience. While more experiments need to be performed to determine the generality as well as the specific nature of the present observations, the findings nonetheless demonstrate that experience-induced alterations to hippocampus circuit function are widespread but not global; they are instead pathway and compartment specific.

## Synthesis

Reviewing Editor: Liset Menendez de la Prida, Instituto Cajal CSIC Decisions are customarily a result of the Reviewing Editor and the peer reviewers coming together and discussing their recommendations until a consensus is reached. When revisions are invited, a fact-based synthesis statement explaining their decision and outlining what is needed to prepare a revision will be listed below. The following reviewer(s) agreed to reveal their identity: Agnès Gruart.

Your ms was reviewed again and we all agree you have addressed most of the previous conerns. There are some remaining points that may require some additional changes. In particular, we feel CSD analysis and results require some clarification.

First the results seem to show some trends which are, however, not statistically significant. For example, Fig. 3C and D show very similar trends of differences for pre- and post-spike but with different statistics and conclusions. Also in new Fig. 4B and D there are large differences, in particular for the slope, but the statistics is negative. We wonder whether the lack of significance is due to the relatively low number of samples? Would the conclusions be different for larger samples? Please address this point carefully.

Second, we feel interpretation/explanation of CSD may be partially misleading in particular regarding physiological relevance. We agree CSD analysis helps to localize inward and outward transmembrane currents as sinks and sources, but it fails to inform on their active (e.g. corresponding to the location of the generator) or passive nature (e.g. the return current through membrane leak and capacitance). To identify these one needs to consider the structure of the network and the location of stimulating electrodes. In this context, the slope of the fEPSP and the pre-spike AUC is likely to dominantly reflect the excitatory synaptic current in the molecular layer, whereas the same parameters measured in the granule cell layer may be primarily reflecting the corresponding passive source. This should be clarified. It is also not clear what advantage pre-spike AUC offers over the slope measurement. Second, the PS may not be a direct measure of the excitability, but regarded more specifically as reflection of the size of the synchronously discharging neuronal subpopulation. Instead, E-S coupling is regarded as a correlate of excitability of the neuronal population - whereby both intrinsic mechanisms and the balance of excitation and inhibition may play an (indistinguishable) role. In fact, the authors refer to both these parameters in their Discussion and arrive to conflicting conclusions (see e.g. page 16, lines 355-356 vs. 360-362). PLease, address this point carefully in your revision.

Finally, the term “post-population spiking activity” (line 157) may be cryptic. It is inconsistently used in the text, and its physiological relevance remains undefined. Traditionally, it is viewed as reflection of inhibition overcoming the brief early excitation, which after the PS is likely to be dominated by feedback inhibition. The authors do briefly refer to feedback inhibition in their discussion, but in a more broader context and conclude that it is unchanged as paired-pulse ratios are unchanged. Paired-pulse ratio, however, is a complex measure which reflects the dynamics and balance of excitation and inhibition, but not well suited to monitor the overall level of and change in feedback inhibition across different animals and experimental conditions.

We feel this lack of definition and interpretation requires further clarification in a revised version.

## Author Response

Responses to reviewers

In what follows, we reproduce the reviewers comments and respond to it.

Reviewers: First the results seem to show some trends which are, however, not statistically significant. For example, Fig. 3C and D show very similar trends of differences for pre- and post-spike but with different statistics and conclusions. Also in new Fig. 4B and D there are large differences, in particular for the slope, but the statistics is negative. We wonder whether the lack of significance is due to the relatively low number of samples? Would the conclusions be different for larger samples? Please address this point carefully.

Response: The reviewers point out that in Fig 3C, the pre-spike sink in the molecular layer looks different for the trained group but does not reach significance, while using the I-O curves in Fig 3D we find that the groups differ.

The difference between reaching significance by analyzing the IO curve (Fig 3D) vs. the single stimulation intensity (Fig 3C) is due to the greater sensitivity of the repeated measures across the stimulus range, compared to the single measurement at the maximum stimulus intensity we evaluated. The revised manuscript now states at line 329 “We evaluated the input-output curves of pre- and post-spiking responses (sl-AUC) at the molecular layer (Figure 3D), both of which show a significant effect of group due to greater statistical sensitivity of repeating the measurements for different stimulus intensities.”

Our intention with Fig 3D is to provide the reader with a sense of where changes may be occurring without the complexity of providing I-O curves at each site. In the revision we state on line 313 “To get an initial sense of where training-induced changes in the evoked response might occur, we quantified the source-localized activity prior to and after the population spike activity by measuring the AUC of the source-localized signal (sl-AUC) in response to 800 μA stimulation, which is the maximum stimulation intensity that was recorded for all animals (Figure 3C).”

The reviewers also questioned whether the lack of significance in Fig. 3D and also Fig 4B and D is due to low sample size. We conducted a power analysis to evaluate the sample size needed to find a significant difference given the design and observed effect sizes (h2). For an 80% chance of finding true significance, atypically large sample sizes would be required. For Fig. 3D: 16 animals/group (total of 48 animals), and for Figures 4B and D: 18-27 animals/group (total of 54 to 81 animals). To help readers appreciate this, we include the effect size in reports of the non-significant statistics and state in the revised methods line 212 “The effect size h2 is given when a non-significant statistical trend is observed.”

Finally, we state on revised line 357 “Although the slopes of the E-S coupling appear different (Figure 4B,D, right panel), significance is not reached, likely due to the small effect sizes. Indeed, a power analysis with power set to 0.80, indicates 3-4 times larger sample sizes are needed to detect group differences in the E-S coupling.”

Reviewers: Second, we feel interpretation/explanation of CSD may be partially misleading in particular regarding physiological relevance. We agree CSD analysis helps to localize inward and outward transmembrane currents as sinks and sources, but it fails to inform on their active (e.g. corresponding to the location of the generator) or passive nature (e.g. the return current through membrane leak and capacitance). To identify these one needs to consider the structure of the network and the location of stimulating electrodes. In this context, the slope of the fEPSP and the pre-spike AUC is likely to dominantly reflect the excitatory synaptic current in the molecular layer, whereas the same parameters measured in the granule cell layer may be primarily reflecting the corresponding passive source. This should be clarified. It is also not clear what advantage pre-spike AUC offers over the slope measurement.

Response: The reviewers ask that we clarify our interpretation of the sinks and sources from the CSD analysis. We agree that an interpretation of the CSD currents would help the reader to understand the figures and measurements and we agree with the interpretation given by the reviewers. In the revision we point out at line 159 of Methods “We note that interpreting an (inward) current sink or (outward) current source depends on knowing the functional anatomy of the relevant sites because active sinks and sources are associated with passive return sources and sink currents, respectively.”

To avoid misleading the reader and to clarify why we use the pre-spike AUC measurement, we provide the number of clarifications in the revised Results that are reported below. The AUC from the CSD trace is useful for evaluating the otherwise difficult to evaluate post-spike activity and because we provide that analysis, we included the pre-spike AUC. By demonstrating that conclusions based on pre-spike AUC (a non-standard measure) are validated by fEPSP slope (the standard measure) we believe the reader can have a better intuition and confidence in interpreting the postspike AUC measures.

Line 291 “We registered the data to the somatodendritic locations and then calculated group average CSD traces (Figure 3B), which appear different, although we did not further analyze the spatial profile of the CSD.”

Line 300 “The second approach evaluates the evoked activity localized to a single electrode at a specific location along the somatodendritic axis. Without relying on assumptions about the waveform of the response, we measured the area-under-thecurve (AUC) of the CSD trace at this site. The activity following spiking is particularly difficult to quantify using traditional methods but can be captured in a straightforward manner by measuring the AUC of the response that occurs after the spike activity. Whether this activity corresponds to active transmembrane currents or passive return currents cannot be determined from the CSD alone and requires knowledge of the functional anatomy for a complete interpretation, and we therefore analyzed the CSD source and sink components separately. In contrast, the activity preceding the population spike standardly estimated by the slope of the fEPSP can also be estimated by measuring the AUC before the PS, which quantifies the entire response instead of the response at a specific time point; these two estimates of the synaptic response at the molecular layer are correlated in our data set (r = 0.87, p = 10-57).” Line 325 “Although the pre-spike molecular layer sink represents the perforant path input, it is uncertain to what extent this difference in the post-spike molecular layer sink reflects a training-induced difference in either an active transmembrane current or a passive return current.”

Reviewers: Second, the PS may not be a direct measure of the excitability, but regarded more specifically as reflection of the size of the synchronously discharging neuronal subpopulation. Instead, E-S coupling is regarded as a correlate of excitability of the neuronal population - whereby both intrinsic mechanisms and the balance of excitation and inhibition may play an (indistinguishable) role. In fact, the authors refer to both these parameters in their Discussion and arrive to conflicting conclusions (see e.g. page 16, lines 355-356 vs. 360-362). PLease, address this point carefully in your revision.

Response: The reviewers ask that we clarify our interpretation of the PS and E-S coupling, pointing out the limitations. Thanks for calling our attention to this.

In the revision we interpret the PS as a measure of synchronous neuronal discharge.

Line 339 “Traditionally, the early slope of the field excitatory post-synaptic potential (fEPSP) is used to estimate the synaptic response and the PS amplitude estimates synchronous neuronal discharge.”

Line 382 “While the conditioning is sufficient to alter population synaptic function, it also results in increased neuronal discharge, evidenced as an increased population spike response at the granule cell layer, whereas the enhanced sl-fEPSP at the granule cell layer is harder to interpret (Fig. 3F) because the PP terminates at the molecular layer.”

Line 392 “An enhanced synaptic component of the evoked response was only observed in the Conditioned rats, whereas compared to Home-Cage rats, the measures of neuronal discharge and post-spike activity were enhanced after either the conditioned or non-conditioned experience.”

Line 399 “The observed increases in sl-PS amplitude in the Conditioned and No-shock groups compared to the Home-Cage group appears not to be due to increased intrinsic excitability as no significant effect of experience on E-S coupling is observed. However, E-S coupling reflects both intrinsic excitability and the balance of excitation/inhibition (Lu et al., 2000; Daoudal et al., 2002), which cannot be distinguished without further experiments.”

Reviewers: Finally, the term “post-population spiking activity” (line 157) may be cryptic. It is inconsistently used in the text, and its physiological relevance remains undefined. Traditionally, it is viewed as reflection of inhibition overcoming the brief early excitation, which after the PS is likely to be dominated by feedback inhibition.

Response: The reviewers ask that we change our use of the term “post-population spiking activity.” We simplified the term to “post-spike activity” and point out that it is a complex and uncertain mixture of activities. Line 156 “Analysis of the individual sources and sinks within the CSD profile allowed us to quantify specific components of the response, such as the post-spike activity, that is otherwise difficult to identify and measure as it is comprised of excitatory and inhibitory responses.”

Reviewers: The authors do briefly refer to feedback inhibition in their discussion, but in a more broader context and conclude that it is unchanged as paired-pulse ratios are unchanged. Paired-pulse ratio, however, is a complex measure which reflects the dynamics and balance of excitation and inhibition, but not well suited to monitor the overall level of and change in feedback inhibition across different animals and experimental conditions.

We feel this lack of definition and interpretation requires further clarification in a revised version.

Response: The reviewers ask that we define and interpret what we are measuring with the pairedpulse ratio. We agree with the reviewers and in fact have found in a separate study we are conducting, a large training-induced change of inhibition. In the revision we added clarifying statements to the Results and Discussion. Line 360 “Finally, the differences in sl-fEPSP and sl-PS cannot not be easily attributed to changes in feedback inhibition, as there is no difference in paired-pulse inhibition measured as the ratio sl-PS2/sl-PS1 (Group: F2,113 = 0.13, p = 0.88; ISI: F1,113 = 2.73, p = 0.10; Interaction: F2,113 = 0.49, p = 0.61; data not shown). Nonetheless, other estimates of altered inhibition merit assessing (Ruediger et al., 2011).”

Line 403 “Alternatively, either altered feedback or feedforward inhibition could account for the observations, but although the paired-pulse paradigm used in these studies did not detect a group difference, other estimates of feedback inhibition are warranted before concluding that feedback inhibition is unchanged by active place avoidance training.”
